# Mycotoxins in Ready-to-Eat Foods: Regulatory Challenges and Modern Detection Methods

**DOI:** 10.3390/toxics13060485

**Published:** 2025-06-09

**Authors:** Eleonora Di Salvo, Giovanni Bartolomeo, Rossella Vadalà, Rosaria Costa, Nicola Cicero

**Affiliations:** 1 Department of Biomedical, Dental, Morphological and Functional Imaging Sciences (BIOMORF), University of Messina, 98168 Messina, Italy; edisalvo@unime.it (E.D.S.); gbartolomeo@unime.it (G.B.); rossella.vadala@unime.it (R.V.); rosaria.costa@unime.it (R.C.); 2 Science4life S.r.l. Start Up, 98168 Messina, Italy; 3 National Research Council, Institute for Agriculture and Forestry Systems in the Mediterranean, 95128 Catania, Italy

**Keywords:** mycotoxins, ready-to-eat foods, fungi, human health, food safety, mycotoxin legislation, artificial intelligence

## Abstract

Mycotoxins are a large family of secondary metabolites produced by filamentous fungi species that may be present in food following fungal growth. Mycotoxins are found in a variety of crops, including wheat, millet, maize, sorghum, peanut, soybean, and their by-products. In recent years, the consumption of ready-to-eat food (RTE) has increased exponentially. An increasing number of consumers have elected to purchase and consume ready-made meals, a choice that allows for a more expedient and convenient dining experience. The aim of this review was to investigate recent literature to find a link between the consumption of mycotoxin-contaminated RTEs, modern detection methods (artificial intelligence), and potential health risks to consumers. The regular exchange of information between the Member States and the European Community (EU) concerning the monitoring of contaminants and undesirable chemical substances, and the subsequent communication of the findings to the EFSA, provides the foundation for the evolution of the legislative framework with the objective of enhancing food safety and reducing the risks associated with the consumption of food. It is imperative that governments, the food industry, and the scientific community collaborate to reduce this risk and ensure consumer safety.

## 1. Background

Mycotoxins are a large family of secondary metabolites produced by filamentous fungi species that may be present in food following fungal growth. The ingestion of mycotoxins by humans, which occurs mainly through plant-based foods and the residues and metabolites present in animal-derived foods, may result in poor liver and kidney function. Moreover, mycotoxins are exogenous compounds with low molecular weight, defined as xenobiotics or contaminants of natural origin. These toxic substances have the potential to cause a range of adverse effects on human and animal health [1]. The genera of fungi and mycotoxin producers that have been most extensively studied are *Penicillium*, *Aspergillus*, and *Fusarium*.

The major mycotoxins of food safety importance include aflatoxins (AFs), fumonisin (FUM), ochratoxin A (OTA), citrinin (CIT), deoxynivalenol (DON), zearalenone (ZEN), and *Alternaria*.

**Figure 1:**
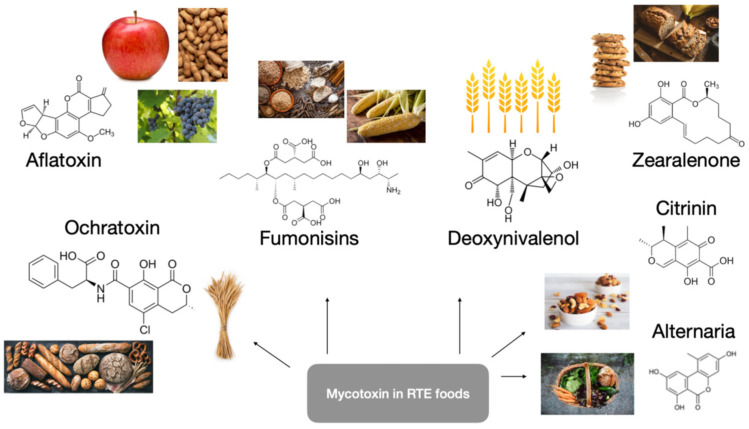
Molecular structures of mycotoxins and correlated food sources.

These mycotoxins could be found in foodstuffs in significant quantities, with implications for human and animal health [2]. Mycotoxins can proliferate on many agricultural and food products. Humans are most exposed to mycotoxins through contaminated cereals, cereal-based products, and ready-made food items derived from animals that have been exposed to them. The contamination of food products may occur at any stage of the food supply chain, from the pre-harvest to the post-harvest stages [3]. The growth of fungi is not necessarily associated with the formation of mycotoxins, and because of their stability, they may be present in food in the absence of fungi. Therefore, when the environment was most favorable for further fungal growth, the fungi used a mycotoxin to multiply [4]. There are many known mycotoxins, synthesized by a variety of fungi, that can be potentially found in different types of food [5]. Mycotoxins are found in a variety of crops, including wheat, millet, maize, sorghum, peanut, soybean, and their by-products (Figure 1). Additionally, cereals, oilseeds, cottonseed, leguminous seeds, spices, and other foodstuffs used for animal feed may also contain mycotoxins [6].

The food industry has created a variety of ready-to-eat (RTE) foods, which are defined as food items that have been prepared by the producer for direct consumption without the necessity for additional preparation or processing [7].

In recent years, the consumption of RTE foods has increased exponentially. An increasing number of consumers have elected to purchase and consume ready-made meals, a choice that allows for a more expedient and convenient dining experience. This surge in the use of RTE by families has compelled the food industry to meet the market demand and to satisfy consumers’ need for convenience [8]. The issue with RTE food is that it may contain a number of elements, including drugs, pesticides, and other substances used in primary production, as well as toxic components created through the processing or storage of the food, such as mycotoxins [9].

The objective of the present review is to examine recent literature to determine whether there is a link between consuming ready-to-eat food contaminated with mycotoxins and health risks for consumers. Furthermore, the review examined the development of artificial intelligence in the context of mycotoxin contamination in recent years. This investigation was prompted by a lack of data on the presence of certain mycotoxins in specific ready-to-eat (RTE) foods and their by-product categories.

## 2. Ochratoxin A (OTA)

Three distinct types of ochratoxin, designated A, B, and C, are classified according to their molecular structure. Nevertheless, OTA is the most toxic metabolite, due to its high abundance and the fact that it causes a range of adverse effects, including nephrotoxic, immunotoxic, hepatotoxic, genotoxic, and teratogenic effects [1,10]. The occurrence of OTA is a spontaneous phenomenon that follows contamination by fungi in a diverse range of foods and beverages, including fruits, cereals, cheeses, coffee, and spices [11]. Until recently, food contamination by OTA was thought to depend only on *P. verrucosum* and *A. ochraceus*, which can contaminate cereals and preserved dried foods. However, in the last decade, it has been discovered that contamination of foods, such as wine, grapes, dried fruits, cereals, and coffee [1], also occurs with species belonging to the black *Aspergillus*, such as *A. niger* aggregate, *A. carbonarius*, and *A. ochraceus*, and with some *Pennicillium* species, such as *P. griseofulvum*, *P. chrysogenum*, and *P. nalgiovense*, that are used as starters in the food industry or isolated from cured meat production [10]. The fungi most frequently isolated from meat or ripened cheese belong to the genus *Penicillium*, a known producer of penicillin. The presence of penicillin in food and feed is undesirable because it may favor allergic reactions or may create antibiotic resistance in humans due to the presence of pathogenic bacteria [12]. *Penicillium* species that have been used as starters in the food industry or isolated from cured meat production are *P. griseofulvum*, *P. chrysogenum*, and *P. nalgiovense*. In particular, *P. griseofulvum* synthesizes four mycotoxins: patulin, cyclopiazonic acid, roquefortin C, and griseofulvin [13]. Several authors have reported that contamination by the *Penicillium* genus is most common in RTE meat. Darwish et al. [14] described mycotoxin contamination, especially of *Aspergillus* and *Penicillium* species, which are important contributors to potent mycotoxins, in 13 samples analyzed from four meat products. Similarly, the presence of AF and OTA in corn, rice, and corn products from three districts (Faisalabad, Sahiwal, and Gujranwala) in Pakistan was detected. Of particular concern is the data indicating that OTA contamination exceeded permitted limits in all samples analyzed, with the highest levels observed in the rice samples [15]. Likewise, Makun et al. [16] identified 107 out of 109 analyzed samples of major Nigerian food and feed, including millet, maize, sesame, fonio, and sorghum, to be contaminated by OTA at levels of 5 μg/kg. These levels have been deemed unsafe by humans and animals since they exceeded permitted limits. In another study, Makun et al. evaluated mycotoxins in rice samples cultivated in Nigeria. The authors confirmed the presence of mycotoxins, including AFs (B1, B2, G1, G2), OTA, and FUM. However, only the OTA concentration was found to be of concern with regard to human health [16].

In a similar research about the findings of other authors, Makinde et al. demonstrated the presence of mycotoxins in RTE corn-based foods sold in non-industrial packaging, where operators may have poorer personal hygiene than those working with dried foods and industrial packaging (e.g., biscuits). The contamination of OTA was reported only in maize-based RTE foods, which were probably processed from wheat. This finding is consistent with the hypothesis that mycotoxin contamination is crop-specific [17].

The highest incidence of OTA contamination has been reported in cereals. Indeed, a study was conducted in Italy on cereals produced in the region Puglia [18]. The results of this study indicated that only a negligible proportion of cereal samples exhibited an OTA concentration exceeding the EU legal limit.

A worrying phenomenon has been observed in food products exported from Turkey and Algeria [19]. In these products, the presence of mycotoxins, including AFs and OTA, was identified. A total of 68.2% of samples of food products exported from Turkey, particularly to Europe, were contaminated with mycotoxins. These products include nuts, cereals and their derivatives, pulses, dried fruits, dried vine fruits, and coffee.

## 3. Aflatoxin (AFs)

The most important mycotoxin of the *Aspergillus* genus is aflatoxin, because of its dramatic implications in food safety due to both its wide distribution in food and feed and its poisonousness. Aflatoxin is a potent toxin produced by the fungi *Aspergillus flavus* and *Aspergillus parasiticus*, which are mainly found in geographical areas characterized by hot and humid climates. Peanuts, maize, wheat, and their derivatives are the main sources of aflatoxin [20]. In recent years, the World Health Organization (WHO) has identified aflatoxins as a significant contributor to the global burden of foodborne diseases. The best studied aflatoxin is aflatoxin B1 (AFB1), which is formed during the first metabolic pathway of AFB1 via the action of the microsomal cytochrome enzyme (CYP450). AFB1 is currently recognized as the most genotoxic and immunotoxic aflatoxin and is responsible for oxidative stress [21]. The EU limit for AFB1 in peanuts and peanut products intended for direct human consumption is 2 μg/kg. Ezekiel et al. [22] reported an increase in mycotoxins in RTE foods and the raw materials used to prepare these foods in Nigeria. In particular, they found a significant increase in AFB1 in RTE foods with low moisture content. In addition, the authors highlighted the presence of mycotoxins, particularly aflatoxins, in granola, a cereal commonly consumed for breakfast by the Nigerian population [22]. Aflatoxins were present in 20% of the samples analyzed, and the European Food Safety Authority (EFSA) set a level of nephrotoxicity in humans at 0.2 μg/kg [23]. Furthermore, the risk to human safety was determined by various parameters such as the high moisture content of the food, the fact that these products are not sold in appropriate packaging (e.g., garri), and the fact that they have not undergone any pre-processing (cooking or frying) [24]. Taken together, these factors could increase the risk of mycotoxin contamination and act as a vehicle for fungal spores in other foods.

In a separate study, other authors analyzed foods regularly sold in Brazilian supermarkets and found AFB1 in foods such as granola, muesli, and cereals. Although the results obtained from packaged products were within legislative limits, the contamination of AFB1 likely occurred due to the use of previously contaminated raw materials [25].

A study of nuts from three different regions of Brazil [26] revealed that the majority of the samples analyzed exhibited low levels of aflatoxins, while only one type exhibited high levels of contamination. The authors hypothesized that the contamination of the latter samples may occur because these nuts were sold in their raw, unshelled state, in contrast to the other two samples. Indeed, shelled nuts contain a greater proportion of water, rendering them more susceptible to contamination and fungal growth.

Further estimation of aflatoxin levels in RTE nuts, including groundnuts, almonds, pistachios, walnuts, and cashews, yielded comparable results, indicating that aflatoxin levels were within the acceptable limit. In particular, this research has demonstrated that the enzyme-linked immunosorbent assay (ELISA) was more sensitive than the thin-layer chromatography method (TLC) for detecting AFB1 in walnut and almond samples. This was evidenced by the positive results obtained using the ELISA method, which were not observed using the TLC method. Consequently, it can be concluded that the ELISA method is more sensitive than the TLC method for detecting mycotoxins [27].

In a preliminary investigation of peanut-based ready-to-use therapeutic foods, a nutritional supplement used for the treatment of AIDS patients and malnourished children, all samples tested exhibited aflatoxin levels exceeding the maximum tolerable level for dietary foods intended for health purposes. Furthermore, the concentration of aflatoxins in locally processed peanut butter is deemed unfit for human consumption according to prevailing global regulations [28].

In accordance with the findings of Carballo et al., the concentration of mycotoxins in RTE food samples was significantly lower than that reported for unprocessed cereals, vegetables, and legumes. In fact, the authors proposed that the risk of mycotoxin contamination was significantly higher in raw food than in pre-treated RTE food, such as fish and meat dishes, given that the latter undergoes various culinary processes [9].

A number of studies have demonstrated that storage time, incorrect temperatures, pH, moisture, and unsuitable packaging can have a negative effect on the safety of raw materials used in RTE foods [29,30]. Furthermore, a study analyzing samples of whole discolored kernels present in RTE products revealed that the risk of contamination by aflatoxins (up to 1383.4 μg/g) is approximately 90%. Although RTE foods undergo peanut sorting processes, it is not always possible to perform sorting procedures that could have removed the discolored kernels (DKs). In light of these observations, the authors concluded that peanut-based RTE foods represent a significant threat to the people of India [31].

Noteworthy are also the levels of AFs in peanuts and peanut butter from Zimbabwe, which reported a significant level (247 ng/g) of total aflatoxins [32]. The contaminated peanut butter samples (91%) exhibited levels of AFB1 that exceeded the maximum permitted levels established by the EU (2 μg/kg) and the Zimbabwean legislation (5 μg/kg). The presence of AFB1 was identified in all of the contaminated samples, with the highest toxin levels observed (55.73 ng/g). Furthermore, another study using high-performance liquid chromatography (HPLC) analysis reported elevated levels of aflatoxins (up to 853 μg/kg) in peanut butter sourced from Sudan [33]. The data reported are alarming in consideration of the highest levels of mycotoxins detected in foods for infants, who are the most vulnerable consumers. For this reason, a number of RTE foods from Pakistan were analyzed, including noodles, cereals, baby food, cream rice, biscuits, wheat, Chinese fried rice, milk powder, gram flour, barley, and peanuts [34]. All positive samples were demonstrated to contain high aflatoxin levels above the EU limit.

## 4. Fumonisins (FUM)

FUMs are produced by *Fusarium* species, with *Fusarium verticillioides* and *Fusarium proliferatum* being the particular species responsible for their production [35]. To date, 15 distinct FUMs have been identified and categorized according to their molecular structure into A, B, C, and P groups. The most prevalent and extensively researched toxin is fumonisin B1 (FB1) [36]. Given the striking similarity in the molecular structure of the FUM, their toxicity can be considered equivalent to that of FB1 at equivalent concentrations. FUMs are commonly present in corn globally, although their toxicity in this food commodity is relatively low and can be regarded as marginal [37]. It is noteworthy that these mycotoxins are present in a wide variety of foodstuffs. However, the adverse effects on human and animal health are only associated with the consumption of corn and other corn-based products [35]. Experimental studies have also demonstrated the renal and hepatic toxicity of FUMs and their potential to cause liver cancer [38]. Epidemiological studies have identified a correlation between the consumption of fumonisin-contaminated corn and the elevated prevalence of human esophageal tumors [39].

In their study, Sbardelotto Di Domenico et al. assessed the concentrations of *Fusarium* and *Aspergillus* in maize samples during storage [40]. The researchers employed four distinct storage methods and monitored the presence of mycotoxins and wheat quality. The findings revealed that airtight bags exhibited the lowest moisture, ash, and lipid contents in wheat, indicating that this storage method effectively limited fungal proliferation.

In another study, Ezekiel et al. sampled 40 flour and grain products and their corresponding cooked foods to investigate the alterations in toxic metabolite profiles between uncooked and cooked foods [41]. As anticipated, mycotoxin concentrations were significantly higher in the flour samples than in the RTE food. Indeed, the cooking process and dilution with water resulted in a 48% reduction in the number of detectable mycotoxins in RTE food samples. Furthermore, the mean concentrations of AFs B1 and FUMs B1 (FB1), FB2, and FB3 were higher in the flour samples than in the RTE food samples.

In another study, 208 corn-based food products from Brazil were analyzed. The results of these analyses confirmed the presence of FUM in corn products, particularly dried corn. The findings of the exposure assessment indicated that certain members of the population, such as those consuming large amounts of corn products and children, may be at an elevated risk of developing corn products [42].

## 5. Zearalenone (ZEN) and Deoxynivalenol (DON)

ZEN is a mycotoxin produced by numerous species of the fungus *Fusarium*, including *Fusarium graminearum* and *Fusarium culmorum*. ZEN is commonly present in all main cereal grains globally because of the effects of fungal infection and growth. However, it can also be found in other crops, such as sorghum, wheat, rye, and barley [43,44]. Toxic effects have been identified in both animals and humans, with pigs identified as the most sensitive species. ZEN is a distinctive mycotoxin with estrogenic activity, capable of modulating the endocrine hypothalamus–pituitary–ovarian estrogen axis [44]. The toxicity of ZEN is attributed to its estrogenic activity, which can manifest as hyperestrogenism, presenting with symptoms such as pseudopregnancy, infertility, stillbirth, abnormal lactation, abortion, and vaginal or rectal prolapse. Endocrine modulation is primarily attributed to altered hormonal equilibrium, which has the potential to influence carcinogenesis in endocrine-sensitive tissues, such as the breast, adrenal glands, prostate, and thyroid [45].

Trichothecenes constitute a group of approximately 180 structurally associated sesquiterpenoid mycotoxins produced by various fungi, including *Fusarium* and *Stachybotrys* [46]. Trichothecenes are classified into four groups (A–D) based on their distinctive functional groups. Group A includes HT-2 and T-2 toxins, whereas Group B comprises deoxynivalenol (DON), 3-acetyl-DON, 15-acetyl-DON, DON-3G, and fusarenon X [47]. Group B mycotoxins have been the subjects of extensive research due to their potential to pose a significant threat to both public health and agro-food systems on both a global and regional scale [48]. The European Commission has recently established a maximum value for DON and the possibility of setting maximum levels for the sum of DON, 3-Ac-DON, 15-Ac-DON, and DON-3-glucoside at 1 μg/kg body weight per day [49]. DON is a toxin primarily produced by *Fusarium graminearum*. This water-soluble toxin, also known as vomitoxin, induces emesis and anorexia in nonruminant animals. The T-2 toxins, widely regarded as toxic members of the trichothecene family, exert potent inhibitory effects on mitochondrial function and protein synthesis. As a result, exposure to T-2 toxins exerts immunosuppressive and cytotoxic effects. Ingestion of trichothecenes causes gastroenteritis, vomiting, and diarrhea. The contamination of agricultural staples, including wheat, barley, and maize, with trichothecenes during the colonization of Fusarium is becoming increasingly prevalent [50]. This trend is due to the expansion of improper processing of agricultural land practices and the influence of climate change [46].

Li et al. examined the contamination of ZEN and deoxynivalenol (DON) in RTE grain-processed products in a region of China. The results demonstrated that among the 50 samples, the detection rates of DON and ZEN were 90.0% and 76.0%, respectively. However, no statistically significant difference was observed in the DON exceeding rate between snack and staple cereal products. A statistically significant difference was evident in the ZEN exceeding rate. This indicates the presence of DON and ZEN in RTE cereal products in specific regions of China, with DON contamination being more prevalent [51].

A novel analytical extraction method proposed by Narváez et al. was validated for the concurrent detection of 18 mycotoxins in RTE pistachios, walnuts, and almonds sourced from the Italian market. The data corroborated the presence of mycotoxins and their metabolism (*Alternaria* metabolites) and the co-occurrence of multiple mycotoxins within the same sample. Overall, the occurrence of copresence was observed in 45% of the walnut samples. In contrast, pistachios and almonds exhibited a lower frequency of co-occurrence, representing up to 27% and 1% of the total samples, respectively. In conclusion, the results demonstrated the considerable prevalence of zearalenone-derived forms and *Alternaria* toxins in RTE nut products, with a notable presence of the ZEN mycotoxin, particularly in pistachio samples [52].

Similarly, Wang et al. conducted a thorough investigation into the occurrence and co-occurrence of mycotoxins in dried fruits and nuts from China, with a particular focus on the measurement of novel mycotoxins that have not been previously reported in China. Moreover, the presence of multiple mycotoxins simultaneously was a common observation in this study. Of the 124 samples that tested positive for contamination, 66 were contaminated with two to eight different mycotoxins. Of these, 34, representing over half of the total positive samples, were contaminated with two different mycotoxins [53].

A number of studies have revealed that the levels of DON decrease with the cleaning of grain, the production of semolina, and the cooking of pasta [54,55,56]. Consequently, cooked pasta retains less than 25% of the original DON content in the grain. In contrast, González-Osnaya et al. [47] proposed that DON contents of up to 623.3 μg/kg could be present. Despite the expectation of a notable reduction in DON content during cooking, the residual concentration of this mycotoxin in ready-to-eat products will remain considerable. Furthermore, DON was identified in 28.0% and 62.6% of the sampled bread and pasta products, respectively. None of the samples exceeded the maximum permitted level of the DON established by the EU for these products. Nevertheless, the data indicated that T-2 toxin was present in 2.7% of bread samples and 9.3% of pasta samples, representing a cause for concern.

## 6. T-2 and HT-2 Toxins

Trichothecenes represent a significant category of mycotoxins, which pose considerable threats to human and animal health, resulting in substantial economic losses for the cereal industry. Trichothecenes constitute a large family of over 200 toxins, which have been detected in a variety of foods, including maize, wheat, rice, walnuts, oats, rye, tomatoes, and barley. These toxins are structurally related to compounds produced by a broad range of species of fungi, such as Fusarium, Trichoderma, Trichothecium, Cephalosporium, Myrothecium, Spicellum, and Stachybotrys [57]. In particular, fusaria species are present in a wide variety of habitats, represent a global problem, and are responsible for the production of the most extensive range of trichothecenes. The most prevalent metabolites that have been identified in agricultural produce include monoacetoxyscirpenol, di-acetoxyscirpenol, neosolaniol, nivalenol, fusarenon-x, HT-2 toxin, and T-2 toxin [58]. In the case of humans, there have been several documented reports of intoxication associated with these trichothecenes. The most common symptoms observed during the initial exposure included gastroenteritis, gastritis, vomiting, diarrhea, and abdominal and esophageal pain. In addition, the following symptoms may be exhibited: excessive salivation, headache, dizziness, weakness, fatigue, tachycardia, fever, and sweating [59].

The EU Commission (2013/165/EU) [60] established legal limits for T-2 and HT-2 toxins, particularly in cereals and their by-products. In contaminated cereal grains, T-2 toxin frequently co-occurs with HT-2 toxin, and in vivo, T-2 toxin undergoes rapid hydrolysis to HT-2 toxin. Consequently, when conducting any potential risk assessment, it is imperative to consider the combination of T-2 and HT-2 toxins. In light of the above considerations, the EFSA has stipulated a group tolerable daily intake (TDI) of 0.1 µg/kg bw for the totality of T-2 and HT-2.

A study was conducted in the southern region of Korea, in which a total of 214 samples were analyzed for T-2 and HT-2 toxins. The samples comprised various types of grains and flour, such as brown rice, barley, and wheat, as well as corn and wheat flour. The analysis was performed using HPLC-FLD detection. T-2 toxin was detected in 11 (5.1%) of all samples. The highest incidence was found in corn (21.7%), followed by mixed grains and brown rice. The presence of HT-2 toxin was identified in 126 (58.9%) of the samples examined [61]. In a different study, T-2 and HT-2 toxins were analyzed in oats (n = 243), oat flakes (n = 529), oat meal (n = 105), and oat by-products (n = 209) from 11 European mills by LC-MS/MS. The limits of quantification (LOQ) were found to be 5 µg kg-1 for both T-2 and HT-2 toxins in oats. The incidence of T-2 and HT-2 toxins (>5 µg/kg) in oats, oat flakes, oat meal, and oat by-products was 93%, 77%, 34%, and 99%, respectively. T-2 and HT-2 were observed to co-occur, with the T-2 level measuring 52% of the HT-2 level in oats [62]. The findings demonstrated an elevated level of T-2 and HT-2 toxins, which exceeded the legal limit established by the EU.

## 7. Citrinin (CIT)

CIT is a polyketide mycotoxin produced by a number of fungal species, including *Aspergillus* (*A. terreus* and *A. niveus*), *Penicillium* (*P. citrinum*, *P. expansum*, and *P. viridicatum*), and the *Monascus* genus. CIT is present in harvested grains and plants, as well as in a wide variety of vegetables and fruits, including beans, spices, herbs, citrus fruits, and spoiled dairy products [63,64]. CIT metabolites have also been produced by the *Monascus* genus, a group of molds that includes *Monascus* purpureus, which is primarily utilized for the fermentation of red rice in East Asian cuisines, particularly in Japanese and Chinese dishes [65]. CIT is most commonly associated with its toxic properties. Indeed, this metabolite induces hepatotoxic, nephrotoxic, carcinogenic, and immunosuppressive effects [64]. Despite the absence of prescriptive regulations pertaining to citrinin contamination, the EFSA has established a threshold of 0.2 μg/kg body weight per day for citrinin, which is deemed to pose no concern regarding nephrotoxicity [66].

A recent study by Ezekiel et al. demonstrated that low moisture RTE foods can harbor diverse fungal species, including AFs and CIT, which pose significant risks at critical levels [22]. Additionally, Somorin et al. provided evidence of the presence of AFs, OTA, and CIT in melon seeds [67]. In particular, the authors demonstrated that the concentration of AFs was significantly higher than the EU limit using HPLC detection. In their analysis of cereal-based products (biscuits, shawarma, and bread), Makinde et al. [68] identified a significantly high concentration of CIT compared with other mycotoxins that could be detected. Moreover, a novel and natural approach, as proposed by Aiko et al. [69], has demonstrated the efficacy of whole claves in inhibiting the proliferation of CIT in white rice. The data indicated that all concentrations of cloves exhibited a dose-dependent inhibitory effect on fungal growth. The authors observed a gradual decrease in the growth of *P. citrinum* with increasing concentrations of clove, noting an inhibition at 1.6 mg/mL using the minimum inhibitory concentration (MIC) method. In rice, the application of clove inhibited fungal growth and CIT production for up to three days.

## 8. Alternaria

*Alternaria* is a genus of fungi that is distributed widely in nature, encompassing both pathogenic and saprophytic species. It has been demonstrated that the *Alternaria* mycotoxins are capable of infecting a wide variety of crops, both in the field and during the postharvest stage. This has been shown to result in significant financial losses due to fruit and vegetable spoilage [70]. The most prevalent Alternaria species comprise *A. alternata*, *A. infectoria*, *A. arborescens*, *A. radicina, A. tenuissima*, *A. brassicae*, and *A. brassicicola*. The organisms under consideration have been found to colonize a range of plants, including cereals, grapes, apples, oranges, lemons, melons, tomatoes, oilseeds, oil crops, cauliflowers, cucumbers, tangerines, and peppers [71]. Furthermore, *Alternaria* species have been shown to produce over 70 toxins, with some of these toxins playing a significant role in the fungal pathogen’s virulence and food safety, given that some of these toxins are toxic to animals and humans [71]. Secondary metabolites of *Alternaria* can be categorized into a variety of chemical groups, including nitrogen-containing compounds (e.g., amides and cyclopeptides), terpenoids, pyranoses, steroids, phenolics, and quinines. *Alternaria* species able to produce secondary metabolites belong to the chemical groups of dibenzo-α-pyrones, such as alternariol (AOH), alternariol monomethyl ether (AME), tenuazonic acid (TeA), altenuene (ALT), and tentoxin (TEN) [72,73]. *Alternaria* mycotoxins are regarded as significant risks to human health due to their potential teratogenic and mutagenic effects in animals. Several in vivo studies have demonstrated the presence of precancerous changes in the mucosa of the esophagus in mice and esophageal cancer in humans. These changes may be associated with the high occurrence of *Alternaria* mycotoxins in cereal grains [74,75]. The EFSA (Commission Recommendation (EU) 2022/553) has established the following limits of quantification (LOQ): 2 μg/kg for AOH and AME in cereal-based foods for infants and young children, and 4 μg/kg for TeA in other foods. The maximum acceptable limit is set at 20 μg/kg for all foods [76]. To date, no universally applicable regulations have been adopted for *Alternaria* mycotoxins, with the exception of the limit of 500 μg/kg for TeA established for sorghum/millet-based infant foodstuffs by the Bavarian Health and Food Safety Authority [77]. Nevertheless, the European Commission has issued a recommendation (EU) 2022/553 on the monitoring of the presence of *Alternaria* toxins in foodstuffs. This recommendation provides guidance on monitoring, but it does not mandate a specific standard method. In this regard, two studies have emphasized the advancement of methodologies for the identification and precise measurement of *Alternaria* mycotoxins [78,79].

Scott et al. observed that both AME and AOH were frequently detected in cereal products from Canada, with concentration levels reaching 9.0 and 4.4 μg/kg, respectively. Furthermore, the presence of TEN was found in 34% of the cereal-based samples (1.5 μg/kg), and TeA was identified in 31% of the samples (124 μg/kg) [80].

In a further study, Lopez et al. documented the presence of notable concentrations of TeA in several food products from the Netherlands, including tomato sauces (462 μg/kg), dried figs (2345 μg/kg), and sunflower seeds (449 μg/kg) [81].

In the most recent research, the authors conducted a comparative analysis of 78 samples of green coffee from all producing regions worldwide. However, contamination of green coffee beans by *Alternaria* toxins was found to be rare and low. Furthermore, the authors reported the mean of *Alternaria* mycotoxins in a variety of food commodities, including cereals and cereal-based products, tree nuts, dried fruits, cocoa, vegetable oil, tomato-based products, spices, herbs, and tea. The authors obtained the LOQs for the following substances: For ALT, the range was from 0.5 to 10 μg/kg; for AOH, from 0.5 to 10 μg/kg; for AME, from 0.5 to 10 μg/kg; for TEN, from 2.5 to 50 μg/kg; and for TeA, the range was from 2.5 to 50 μg/kg [82]. Table 1 presents a summary of data pertaining to the contamination of RTE foods with mycotoxins.

## 9. Mycotoxin Detection

The identification of mycotoxins is contingent upon the extraction method employed, the nature of the preliminary samples, and the specific technique utilized. The prevailing focus in the field of sample preparation at present is on the identification of environmentally sustainable solvents, the streamlining of processes, and the attainment of expeditious outcomes. The most critical steps prior to mycotoxin analysis are the extraction method [102]. The primary objective is the removal of contaminants and mycotoxins from the feed and food samples using an adequate solvent. The selection of appropriate solvents, in conjunction with the extraction method, plays a pivotal role in the efficacy of the process. The selection of an extraction solvent is predicated upon the principle of ensuring that the mycotoxins are removed from the sample with the utmost efficiency. Mixtures of methanol–water and acetonitrile–water at varying ratios are the most frequently employed extraction solvents in mycotoxin analysis [103]. The presence of fatty acids, essential oils, and pigments in the samples renders the extraction process challenging and necessitates the utilization of diverse extraction solvents. For the extraction of mycotoxins, a mixture of ethyl acetate and formic acid [104], 1-octanol and toluene [105], dichloromethane [106], acetone [107], and chloroform [108] have been employed. Hydrophobic mycotoxins have been found to be readily soluble in a range of conventional solvents, while polar mycotoxins, such as fusarins, have been found to be soluble in water. He et al. [109] published a report on using a deep eutectic solvent (DES) for extracting aflatoxins from rice. The extractant was made from two components, tetramethylammonium chloride and malonic acid, which are environmentally friendly, secure, and cost-effective.

The elective techniques for the detection of mycotoxins are high-performance liquid chromatography (HPLC), thin-layer chromatography (TLC), mass spectroscopy, and gas chromatography (GC). In addition to the previously mentioned methods, commercial immunometric assays, such as ELISAs and membrane-based immunoassays, are also employed for the purpose of detection. TLC is a simple and fast technique for the determination of mycotoxins but it is rather dated and especially lacking in terms of sensitivity and accuracy. Because of these limitations, it has been superseded by HPLC for the quantitative analysis of mycotoxins [110]. Technological advancements have progressed from the detection of a single compound to the concurrent detection of multiple targets, utilizing sophisticated composite cleanup stages, such as QuEChERS and solid-phase extraction methods. The latter is particularly advantageous due to its ability to simultaneously extract multiple mycotoxins, its reduced solvent utilization, cost-effectiveness, rapidity, and detection limit that is below the EU regulatory threshold [111]. In recent years, the rapid advancement of detection methods and the introduction of biotechnologies have led to a significant improvement in mycotoxins assessment. New technical methods, including ultrafast performance liquid chromatography (UFLC MS/MS) [112], time-resolved fluorescence immunoassay (TRFIA) [113], nanoparticles used to enhance the traditional ELISA method [114], and lateral flow immunochromatographic assay (LFICA) [115], have been employed in the last few years for the analysis of mycotoxins in feeds, foods, and agronomic goods. These methods offer low cost, simplicity, and speed, which are advantageous for large-scale analysis.

Despite the existence of a multitude of analytical techniques for the detection of mycotoxins, including HPLC and its associated variants (LC-MS/MS), the lack of validated analytical methods represents a significant obstacle to the implementation of standardized monitoring procedures for food and feed products used by humans and animals alike [116]. LC-MS offers a number of advantages over HPLC for the detection and confirmation of trace levels, particularly in the context of complex matrices. Furthermore, it enables the acquisition of data pertaining to the identity of mycotoxins [117]. Moreover, the demonstration of the potential co-occurrence of diverse mycotoxins, their metabolites, and various toxic compounds within a single food product, as well as the identification of novel mycotoxins, necessitates the implementation of sensitive, accurate, and precise analytical protocols [17]. For these reasons, rapid methods should be adopted, including immunochemical methods and improvements to existing techniques like MS/MS. These are optimal due to their speed, cost-effectiveness, and ability to identify and quantify a range of mycotoxins [118]. It is important to note that although the existence of regulations concerning mycotoxin exposure, the efficacy of food safety controls for RTE foods can be further improved. In particular, the implementation of more sensitive detection technologies and more accurate sampling methods are necessary for effective risk management. The current uncertainty regarding human exposure is largely attributable to the interplay of numerous variables and the dearth of experimental data. It is not possible to recommend a single method that is applicable to all types of samples. However, the selection of a method should be based on the type of sample, the research objective, and the laboratory facilities available.

## 10. Mycotoxins Regulations

Mycotoxin regulations vary from country to country and region to region, but most governments have set maximum acceptable levels for mycotoxins in food. Compliance with these regulations is critical for food manufacturers to ensure the safety of their products. Understanding mycotoxin regulation is essential for producing safe RTE foods [119]. The overarching EU strategy is designed to guarantee a superior standard of food safety, welfare, and animal health within the European Union. The strategy employs a unified approach and rigorous monitoring, while simultaneously ensuring the effective operation of the internal market [120]. The regulations pertaining to the implementation of heightened levels of official scrutiny on the importation of specific feedstuffs and foodstuffs of non-animal origin are delineated in Commission Regulation [121]. Furthermore, the European Commission has introduced specific regulations governing the import of foodstuffs from selected third countries due to the potential contamination by aflatoxins. In regard to the regulation of T-2 and HT-2 toxins in cereal products, the European Commission has recently assumed [122] that obliges Member States to monitor these two toxins in foodstuffs. Furthermore, they are required to encourage the simultaneous analysis of samples for the presence of T-2 and HT-2, as well as other fusarium toxins. The legal limits imposed by Regulation (EU) 2023/915 [123] of the EFSA, as reported in the parameters for AFs (2.0 to 12 µg/kg), OTA A (from 2.0 to 80 for spices and liquorice), DON (from 500 to 1.750 µg/kg for wheat and durum wheat grains), ZEN (from 50 to 400 µg/kg for refined oil), and FUM (from 100 to 4000 µg/kg for unprocessed corn grains), are notably high. For these reasons, the modification of the previous regulation by the introduction of new limits for T2 and HT2 toxins (Commission Regulation (EU) 2024/1038) [124] that exclusively refer to cereals and their derivatives and are significantly lower than the permitted limits, has led many authors to identify significantly higher values than those imposed by the regulation. Moreover, it has been demonstrated that the concentration of mycotoxins varies on a yearly, seasonal, and geographical basis. For instance, concentrations of the FUM and *alternaria* mycotoxins appeared to increase from west to east across the Prairies and the province of Quebec. In addition, OTA A exhibited annual cyclic increases in late summer [125]. On the other hand, a distinct legal limit for the detection of mycotoxins is established in each country. It is evident that there is a discrepancy in the legal limits imposed on the presence of mycotoxins in foodstuffs among the producing countries, such as Brazil, the USA, and Canada. In Canadian law, the LOD for mycotoxins varies depending on the specific food type and toxin. However, Health Canada has determined a maximum level (ML) of 15 ppb for total aflatoxins in nuts and nut butters. In addition, various MLs have been proposed for different foodstuffs. For example, 3 ppb has been suggested for grains and 7 ppb for wheat bran. Likewise, in the U.S., the limit of detection for mycotoxins in food, particularly regulated mycotoxins such as AFs, FUM, and ZEN, can vary depending on the specific mycotoxin and the type of food product. The Food and Drug Administration (FDA) [126,127] has established a maximum limit of 20 μg/kg (or 20 ppb) for total AFs in peanuts, a limit of 2000–4000 μg/kg for FUM in maize and maize products, and regulatory limits for DON of 200–500 μg/kg in cereals and cereal products, and a range of 20–100 μg/kg in cereals and cereal products.

In order to address the issue of mycotoxin legislation in Brazil, the LOD for mycotoxins was established. The maximum permitted levels of AFs in milk are 500 nanograms per liter (ng/L), while the levels of DON in finished wheat products intended for human consumption are 1 parts per million (ppm). Limits for other mycotoxins, such as ZEN, can range from 20 to 1.000 micrograms per kilogram (μg/kg), depending on the specific product and region.

The regular exchange of information between the Member States and the European Community concerning the monitoring of contaminants and undesirable chemical substances, and the subsequent communication of the findings to the EFSA, provides the foundation for the evolution of the legislative framework with the objective of enhancing food safety and reducing the risks associated with the consumption of food. It is important to emphasize that these maximum tolerated levels vary considerably among countries, necessitating standardization to eliminate the significant variability in standards. Currently, there is an absence of an international standard for mycotoxins [128]. A multitude of endeavors have been dedicated to the establishment of standards for mycotoxins in food and feed. However, it has proven to be remarkably challenging to achieve a consensus on the maximum levels that should be incorporated within these standards. The major obstacles to achieving a consensus on this matter are the significant variations in contamination levels on a global scale, as well as the divergent capabilities of nations in reducing mycotoxin levels in an economically viable manner.

## 11. Future Perspective

The presence of mycotoxins in many food products, such as RTE foods, represents a significant global public health risk. Mycotoxins continue to represent an important challenge to food safety [129], particularly in developing countries where the majority of the world’s cereal consumption originates and where, moreover, the consumption of RTE products is higher [130]. The adverse consequences of foodborne toxins are exacerbated by the prevalence of poverty and malnutrition in developing countries. Despite the acknowledged risks to human health posed by the consumption of contaminated grain, the practice of feeding contaminated grain to livestock is widespread. This phenomenon exerts a deleterious influence on animal productivity and food supply, while concomitantly contributing to the prevalence of economic disadvantage [131]. In developed countries, mycotoxin risk is effectively managed, though the threat remains significant, particularly in the context of climate change and international trade. The maintenance of elevated standards of control and prevention is imperative for the preservation of public health [132]. The findings of this review indicate that contamination by mycotoxins can occur at any stage of the production chain, from the initial cultivation of raw materials to the processing and packaging of RTE products. Additionally, improper cleaning or inadequate packaging of RTE products contributes to the promotion of fungal growth due to the increased humidity that they cause. The pursuit of efficient and cost-effective solutions has become an increasingly urgent need [17].

Several studies have shown that the procedures involved in the processing and handling of food items such as corn, wheat, granola, and their derivatives, followed by the subsequent storage and cleaning phases, provide an ideal environment for the growth of mycotoxins. The most concerning findings were reported by Kumar et al. [133], who discovered elevated levels of AFs and CIT in RTE cereal-based products and cheese balls, which are commonly consumed by preschool and early school-aged children. A number of studies have indicated the presence of mycotoxins in a variety of foodstuffs, including bread, cereals, snacks, and dried fruit. In many cases, the levels of these toxins are found to be considerably high, which may pose a potential risk to consumers, particularly children, the elderly, and immunosuppressed patients who are more vulnerable to such contaminants [134].

Finally, risk assessment through the “supply chain” is a technique that has been widely used in recent years and could prove beneficial in the conservation of raw materials used in RTE products.

### 11.1. Mycotoxin Prevention and Control

In the present context, the most important finding is the need to prevent fungal growth and mycotoxin formation and to implement strategies to reduce the risk of mycotoxin contamination in RTE products. Achieving this objective necessitates the implementation of novel strategies, including the reduction in available water [135], the optimization of storage conditions [136], the establishment of good agricultural handling practices [137], and the formulation of recommendations for food processing techniques [138]. Post-harvest prevention strategies have been shown to be highly effective in reducing mycotoxins, which are known to be produced during this phase of the food chain. However, it should be noted that pre-harvest natural contamination cannot be completely eliminated. Nevertheless, the application of processing techniques has been demonstrated to minimize subsequent entry into the food and feed chain, thereby reducing the risk of mycotoxin contamination [139]. Furthermore, wet cereals must be dried efficiently and promptly for medium- and long-term storage in hygienic silos, which must be free of insect pests and moldy material. It is also vital to ensure that there is traceability during silo storage and transport for processing. In order to achieve this, it is essential to implement Good Agricultural Practice and operation, as well as to establish approved supplier chains. The use of effective diagnostic tools must be implemented to monitor and quantify mycotoxins at a rapid rate [140]. The issue of representative sampling for stored commodities remains problematic. Although legislation exists pertaining to the sampling protocols, these are challenging to implement, and the discrepancies between the actual sampling and the subsequent analysis of mycotoxin contamination levels may be substantial. The potential exists for the early detection of changes in stored commodities caused by insect or mold activity through the monitoring of intergranular gas composition and the utilization of volatile fingerprints [139]. The development of models on mycotoxigenic mold activity and the conditions that will preclude mycotoxin production, and which can offer an indication of limits relevant to the legislative definition, is of significant importance. Prevention and control of mycotoxin contamination is essential to ensure food safety and reduce the risk of health problems.

### 11.2. The Application of Innovative Materials

The techniques employed for quantitative microbial risk assessment can predict, assist, and intercept the risk of mold contamination. Additionally, for many years, the predominant tool for ensuring the quality of raw foods has been the use of chemical sanitizers, such as chlorine, to control fungal proliferation [141].

Nevertheless, these treatments cannot eliminate the residual deposits formed during the production of foodstuffs. The addition of hypochlorite during treatment may be noxious as well [142]. The use of modified atmosphere packaging (MAP) after harvesting could prove to be a beneficial alternative. Processing and potential sanitizing treatments can extend the shelf life of fresh produce without compromising its organoleptic characteristics. Indeed, the use of diverse packaging technologies can influence factors such as CO_2_ levels or moisture [143]. A significant finding of this review is the necessity for increased awareness among consumers and producers regarding the importance of storage and transportation conditions. This is attributed to the fact that fungal growth and subsequent production of mycotoxins can be intensified by favorable environmental conditions, such as high humidity and high temperatures [144]. Furthermore, it has been suggested that the use of natural compounds, including citrus, cinnamon, and clove oil [145], various essential oils [146], and whole cloves [69] may be effective in inhibiting mycotoxin proliferation in foods. The low solubility and stability, as well as the high cost, of these bioactive substances have made it challenging to use them in food products. Consequently, future studies should focus on investigating the potential interactions between these compounds, foods, and the human body, particularly the gastrointestinal tract [147]. Phytochemicals and natural plant compounds have the potential to play crucial roles in the inhibition of fungal proliferation without altering the intrinsic characteristics of foods, thereby reducing the risk associated with the use of chemical additives [147]. In addition, most mycotoxins remain chemically and thermally stable during various food processing techniques, including boiling, cooking, baking, pasteurization, and roasting. For this reason, the detoxification of mycotoxins is of paramount importance [148].

Mycotoxin detoxification is a multifaceted process that involves several strategies to mitigate the harmful effects of mycotoxins in the body [149]. One effective approach is the use of glutathione, a potent antioxidant that aids in detoxification by neutralizing mycotoxins and other toxins, thereby supporting cellular protection against oxidative stress [150]. In addition, mycotoxin binders such as activated charcoal and cholestyramine can be used to bind mycotoxins in the gastrointestinal tract, preventing their absorption into the bloodstream [151]. These substances facilitate the removal of toxins from the body and enhance natural detoxification processes. In addition, mycotoxin testing is critical for determining exposure levels, allowing for targeted detoxification strategies tailored to individual needs. Together, these methods provide a comprehensive framework for effectively addressing mycotoxin-related health concerns [152]. Moreover, a recent study reported that two extracts from *Aspergillus terreus* and from *Alternaria alternata* exhibited anti-inflammatory activity. This finding was supported by a comprehensive metabolomics analysis of extracts from five endophytic fungi isolated from medicinal plants in Sudan. The authors of the study found that the extracts in question were capable of inhibiting the pro-inflammatory effect of LPS on differentiated THP-1 cells. Furthermore, a combination of standardized analyses, including GC-MS, LC-MS, and HPTLC in conjunction with molecular networking data processing, was employed to facilitate the identification of distinct metabolite structures. These extracts were observed to induce an anti-inflammatory state in the cells, rendering them less susceptible to the pro-inflammatory effect of LPS. This constitutes a novel and significant finding with regard to the reuse of secondary metabolites extracted from mycotoxins in the treatment of inflammatory diseases [153].

## 12. Impact of Artificial Intelligence on Mycotoxin Contamination

Recent years have seen a rapid expansion in the field of artificial intelligence (AI), with significant advancements in various areas, including scientific research fields such as tumor detection, biofilm detection and identification [154,155], electroencephalographic (EEG) analysis [156], and fungi identification [157]. Indeed, AI technologies have the potential to enhance food safety by facilitating advanced detection and monitoring of mycotoxins in human foods [158]. Recent developments in the field of mycology have been marked by a substantial increase in the utilization of AI, as evidenced by noteworthy progress in the identification of fungi and the detection of mycotoxin contamination [159]. As mentioned above, conventional detection analyses such as ELISA, HPLC, LC-MS, and GC-MS produce complicated results that require in-depth interpretation and evaluation. Nevertheless, the risk and interest in mycotoxins have increased significantly in recent years, particularly in 2024.

**Figure 2:**
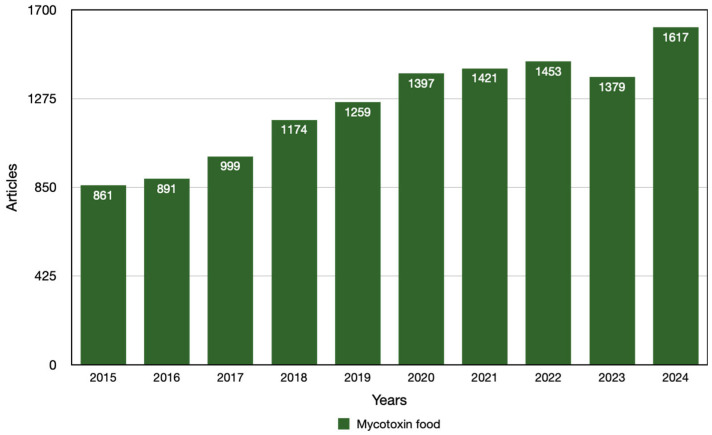
Articles published per year between 2015 and 2024, focusing on mycotoxins in food, found on PubMed using the keywords “mycotoxin food”.

**Figure 3:**
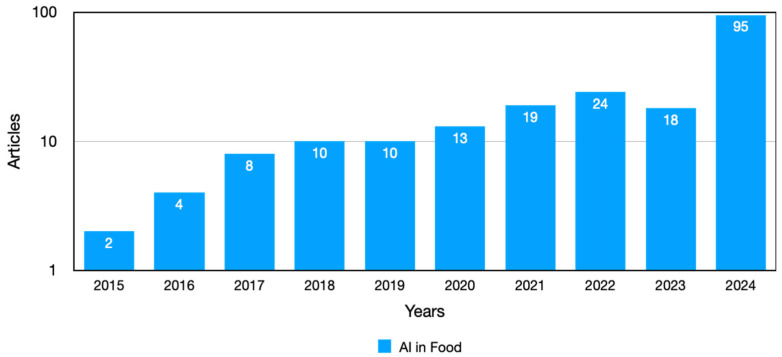
Articles published per year between 2015 and 2024, focusing on AI’s potential to analyze food contamination, found on PubMed using the keywords “Food contamination detection artificial intelligence”.

For these reasons, AI could be considered a valid alternative to conventional detection techniques. Consequently, recent years have seen a surge in the popularity of novel approaches for mycotoxin detection and prediction. The term AI refers to a range of computational methods and techniques that enable computers to evaluate and learn from data, as well as to identify patterns, thereby acquiring the capacity to make decisions that are almost independent of human intervention. Machine learning (ML) techniques, such as supervised learning, neural networks (NNs), support vector machines (SVMs), and random forest (RF), are beginning to demonstrate promise in the processing of complicated datasets created by various analytical methods [160]. Moreover, the combination of AI with a range of analytical methodologies, including spectroscopy, biosensors, and imaging techniques, promises significant advancements. This novel approach aims to enhance the accuracy, efficiency, and rapidity of mycotoxin analysis. Computer vision is a detection technology that shows great promise. The simulator is designed to emulate the human visual system, offering several advantages, such as high detection speeds, low cost, ease of maintenance, and high visualization. As shown in Figure 2 and Figure 3, the trend of mycotoxin contamination has increased over the past decade, with the majority of publications occurring during the 2020–2024 period. This suggests that the utilization of ML algorithms for the detection of mycotoxins in food has undergone significant growth in recent years.

It is evident that several techniques of AI have demonstrated the capacity to detect mycotoxins with a high degree of accuracy and efficiency in a multitude of food products, including wheat, rice, maize, barley, and peanuts [161]. Among the various mycotoxins that have been detected by these techniques are AFs, FUM [161], and DON [157].

The employment of advanced hyperspectral photography, electronic noses, and biosensors, in conjunction with deep AI models, has resulted in a significant enhancement in the efficacy of detection techniques. The continuous advancement of AI and its associated applications has the potential to enhance the accuracy, efficiency, and cost-effectiveness of mycotoxin detection [162].

In the era of rapidly advancing AI technology, a symbiotic relationship between regulatory agencies and academic institutions is imperative for the attainment and preservation of food safety.

In addition, mycotoxin prediction and control are undergoing a significant transformation due to advancements in AI, ML, and deep learning (DL) [163]. These computational models accurately predict fungal proliferation and mycotoxin production in vitro and have been used to classify contaminated fields and foods. The employment of computerized systems has augmented the efficacy and adaptability of these instruments for real-time monitoring.

## 13. Conclusions

The presence of mycotoxins in RTE foods constitutes a real health risk that is the subject of ongoing research and regulatory investigation. Food may be contaminated at various stages of production and handling with mycotoxins, which are toxic compounds produced by certain fungi. The exposure to mycotoxins includes severe and chronic health effects, e.g., carcinogenicity, immunosuppression, and neurotoxicity. In view of the increasing consumption of RTE foods in the modern diet, it is imperative that strict monitoring and control measures are implemented to reduce the contamination with mycotoxins. To determine the prevalence of specific mycotoxins in different food sources, understanding the mechanisms of their toxicity and developing effective detection and decontamination strategies should be the focus of future research. Increased public awareness and education about mycotoxins could also play a central role in consumer safety.

Additionally, in the future perspective, the integration of artificial intelligence, novel technologies, and regulatory legislation has the potential to enhance the accuracy and efficiency of mycotoxin contamination control.

Overall, it is essential to address the issue of mycotoxins in RTE foods in order to protect public health and ensure food safety. This multi-faceted approach will not only protect consumers but also build confidence in food safety standards, ultimately leading to healthier diets and improved public health outcomes.

## Figures and Tables

**Table 1:** Research articles that have investigated the presence of mycotoxins in ready-to-eat food in different regions of the world (from 2011 to 2024).

Country of Origin of Sample	Type of Food	Mycotoxin	Target Analyte Concentration (Mean-μg/kg)	Methodology	Reference
Sudan	Peanut butter	AFs	287 ± 200.5	HPLC	[33]
Nigeria	Rice	OTA	134–341	HPLC	[16]
Spain	Bread	DON	12.2	GC-MS	[47]
Italy	Hazelnut	AFB1	91.7 ± 5.2	HPLC-FLD and HPLC-MS	[19]
		AFB2	74.7 ± 6.7		
		AFG1	89.7 ± 1.7		
		AFG2	60.0 ± 8.8		
		AFBs	86.0 ± 4.1		
	Wheat flour	AFB1	94.0 ± 2.4		
		AFB2	78.0 ± 9.2		
		AFG1	91.0 ± 3.6		
		AFG2	91.0 ± 3.6		
		AFBs	89.4 ± 3.3		
	Dried figs	AFB1	98.2 ± 6.4		
		AFB2	76.0 ± 5.3		
		AFG1	90.8 ± 3.9		
		AFG2	53.3 ± 5.7		
		AFBs	88.5 ± 5.2		
	Coffee	OTA	88.7 ± 5.4		
	Sultanas		99.8 ± 3.5		
Indonesia	Maize kernels	DON	59.9–202	HPLC	[83]
	Fried maize		67.1–348		
	Cereal	AFB1	0.2 ± 0.01		
	Noodles	AFB1	0.40 ± 0.09		
	Baby powder milk	AFB1	0.17 ± 0.05		
		AFB2	0.03 ± 0.01		
		AFG1	0.11 ± 0.03		
		AFG2	0.07 ± 0.001		
	Cream rice	AFB1	0.07 ± 0.02		
Pakistan	Biscuits	AFB1	0.31 ± 0.01	RP-HPLC	[34]
		AFB2	0.38 ± 0.01		
		AFG1	1.13 ± 0.06		
		AFG2	0.68 ± 0.01		
(continued)	Wheat	AFB1	0.28		
	Chinese fried rice	AFG1	0.025		
		AFG2	1.3		
	Milk powder (tea)	AFB1	0.05		
		AFG1	0.15		
		AFG2	0.09		
	Gram flour	AFB1	0.12		
		AFB2	0.59		
		AFG1	0.35		
		AFG2	0.80		
	Barian	AFB1	0.07		
		AFG1	0.15		
		AFG2	0.25		
		AFT	0.48 ± 0.06		
	Peanuts	AFB1	9–71		
Canada	Cereal products	AME	9.0	LC-MS/MS	[80]
		AOH	4.4		
Africa	*Kuli-kuli* (snack)	AFB1	25.5 to 455	ELISA and LC–MS/MS	[84]
Nigeria	Corn-based	AFB1	14.0	TLC	[85]
		AFB2	8.0		
		AFG1	6.0		
	Groundnuts-based	AFB1	8.5		
		AFB2	9.0		
		AFG1	15.8		
	Nut-based wheat-based	AFB1	6.0		
		AFB1	17.8		
		AFG1	13.0		
Nigeria	Peanut cake	AFB1	88.0	TLC	[86]
		(A. flavus)			
		AFB1	8.0		
		(A. parasiticus)			
Kenya	Raw peanuts	AFBs	201	ELISA	[87]
	Roasted coated peanuts	AFBs	382		
Zimbabwe	Peanuts and peanut butter	AFs	75.66 ng/g	HPLC	[32]
Nigeria	Maize grain	AFB1	394	LC-MS/MS	[88]
		AFB2	44		
		AFG1	47		
		AFG2	16		
		AFM1	14.5		
		DON	60		
		FUMB1	1.552		
		FUMB2	442		
		FUMB3	161		
		OTA	111		
		ZEN	174		
Malawi	Peanut butter de-skinned	AFB1	13.2–40.6	IAC-LC	[28]
		AFB2	1.7–7.2		
	Roasted groundnut	AFB1	0.1–12.3		
		AFB2	0.2–1.8		
Nigeria	*Kokoro* (snack)	AFB1	0.75–7.25	ELISA	[89]
Ghana	Ice-kenkey (dessert)	AFB1	7.01–20.54	HPLC	[90]
		AFB2	0.51–1.63		
		AFG1	0.47		
Nigeria	Groundnuts (raw)	AFB1	104.1	TLC	[91]
	Groundnuts (roasted)	AFB1	14.1		
Brazil	Maize	FUM	1.83 ± 13.38	Plate count	[40]
Tunisia Spain	Dried fruit	AFB1	16.5	LC-MS/MS	[92]
		AFB2	1.1		
		AFG1	3.2		
		AFG2	5.5		
		OTA	1.2		
Netherlands	Tomato sauces	TeA	462	LC-MS/MS	[81]
	dried figs		2345		
	Sunflower seeds		449		
Nigeria	*Aadun* (snack)	AFB1	1.08–1.88	HPLC-MS	[93]
		AFB2	0.99–1.98		
		AFG1	0.92–1.23		
		AFG2	0.91–1.73		
United Kingdom	Melon seeds	AFB1	9.7	HPLC-MS/MS	[67]
		AFB2	1.5		
		AFG1	1.3		
		AFG2	0.2		
Cameroon	Maize-fufu	AFB1	0.9 ± 0.4	LC-MS/MS	[94]
		DON	23 ± 7		
		ZEN	49 ± 38		
Nigeria	Garri (flour)	DON	35.0–99.0	LC–MS/MS	[95]
		FUM B1	45.0–80.0		
		FUM B2	29.0–65.0		
		ZEN	11.0–17.0		
Ivory Coast	*Garba* (*couscous*)	AFB1	3.44	HPLC-FLD	[96]
		AFB2	1.90		
		AFG1	8.07		
		AFG2	0.56		
		OTA	0.42		
Nigeria	Roasted cashew nuts	AFs	0.1–6.8	ELISA	[97]
India	Groundnut	AFs	13.834	HPLC	[31]
China	Dry fruits	AFB1	49.47 ± 2.02	HPLC	[53]
		AFB2	0.83 ± 0.02		
		ZEN	20.48 ± 0.34		
		OTA	6.23 ± 0.40		
Sierra Leone	Roasted peanut	AFB1	0.62–1.387	LC-MS/MS	[30]
		AFB2	6.49–271		
		AFG1	0.34–3328		
		AFG2	14.7–742		
		AFM1	1.22–66.8		
Pakistan	Biscuits	OTA	23.9	LC	[15]
	Bread		1.96		
Nigeria	Uncooked flour/grain	AFB1	34	LC-MS/MS	[41]
		FUM	500		
		ZEN	200		
China	Cereal products	ZEN	39.2	ELISA	[51]
		DON	975.0		
Nigeria	Granola	AFB1	30.9	LC-MS/MS	[22]
		AFB2	6.72		
		AFG1	1.42		
		AFM1	2.27		
		CIT	1481		
		DON	158		
		FUM A1	7.23		
		FUM B1	74.0		
		FUM B2	25.2		
		OTA A	3.47		
		ZEN	1.47		
Italy	Almonds	AFB1	0.45	LC-MS/MS	[52]
	Almonds	ZEN	3.70/4.54		
	Walnuts	ZEN	0.93		
	Pistachios	ZEN	0.96/8.6		
Switzerland	Cereal-based	ALT	0.5	LC-MS/MS	[82]
		AOH	0.5		
		AME	0.5		
		TEN	0.5		
		TeA	2.5		
	Green coffee	ALT	2		
		AOH	2		
		AME	2		
		TEN	2		
	Cocoa	TeA	10		
Nigeria	Biscuits	CIT	10.9	LC-MS/MS	[68]
		ZEN	4.0		
		DON	308		
	Bread	CIT	10.2		
		DON	59.8		
	Shawarma (kebab)	CIT	105		
		DON	23.6		
Portugal	Breakfast Cereals	AFB1	194.2 ± 164.0	HPLC-MS/MS	[98]
		AFG2	68 ± 42.8		
		CIT	6.7 ± 9.9		
		FUM B1	178.6 ± 72.5		
		FUM B2	34.9 ± 3.5		
		OTA	8.4 ± 2.6		
		ZEN	65.2 ± 57.6		
	Cereals (infant)	AFB1	0		
		AFG2	0		
		CIT	0		
		FUM B1	41.0 ± 33.5		
		OTA	0		
		ZEN	0		
	Snacks	AFB1	0		
		AFG2	0		
		CIT	0		
		FUM B1	8.8 ± 5.7		
		OTA	0		
		ZEN	0		
From 25 countries	Peanuts	AFB1	4.1	LC-MS/MS	[99]
		AFB2	3.7		
		AFG1	0.4		
	Brazil nuts	FUM B2	2.9		
		DON	0.6		
	Hazelnuts	AFB1	0.6		
		AFG2	0.6		
		FUM B1	13.9		
	Macadamias	AFB1	0.3		
		AFB2	0.2		
		AFG1	0.4		
		AFG2	0.2		
		FUM B1	0.3		
		FUM B2	4.6		
		DON	1.9		
	Almonds	AFB1	0.4		
		AFB2	7.6		
		AFG2	0.3		
	Pecans	AFB2	0.3		
		AFG2	4.0		
		DON	1.1		
	Pine nuts	AFB1	0.7		
		FUM B1	0.3		
		FUM B2	17.8		
		DON	2.6		
	Pistachios	AFG2	0.7		
		FUM B2	2.6		
		DON	0.3		
	Walnuts	AFB1	3.4		
		AFG1	0.2		
		FUM B2	9.4		
		DON	1.3		
Egypt	Basterma (typical dish meat)	OTA	17.26	HPLC	[100]
	Corn flex	OTA	0.55		
Nigeria	Nuts	AFB1	0.1	LC-MS/MS	[101]
		AFB2	0.1		
		AFG1	0.1		
		AFG2	0.1		
		ZEA	3.0		
		DON	6.1		
		FUM B1	7.6		
		FUM B2	7.6		
		OTA	0.5		

## Data Availability

Not applicable.

## Abbreviations

The following abbreviations are used in this manuscript:

RTE	ready-to-eat food
OTA	ochratoxin A
AFs	aflatoxins
FUM	fumonisin
CIT	citrinin
DON	deoxynivalenol
ZEN	zearalenone
LC-MS/MS	liquid chromatography–mass spectrometry
HPLC	high-performance liquid chromatography
FLD	fluorescence detector
TLC	thin-layer chromatography
RP-HPLC	reverse phase HPLC
IAC-LC	immuno-affinity column and reversed-phase liquid chromatography
DAD-HPLC	diode array detector-HPLC
EFSA	European Food Safety Authority
AI	artificial intelligence
